# Amphetamine-Dextroamphetamine-Induced Cardiomyopathy, an Emerging Cause of Heart Failure in Young Patient Populations: A Case Study Involving the Study Drug

**DOI:** 10.7759/cureus.40942

**Published:** 2023-06-25

**Authors:** Jennaire T Lewars, Karen P Wiarda

**Affiliations:** 1 Internal Medicine, Carle Foundation Hospital, Urbana, USA; 2 Internal Medicine, Saint James School of Medicine, Chicago, USA; 3 Cardiology, Carle Foundation Hospital, Mattoon, USA

**Keywords:** amphetamine-induced cardiomyopathy, cardiomyopathy, medication overuse, narcolepsy treatment, prescribed adderall, amphetamine-dextroamphetamine

## Abstract

Amphetamine-Dextroamphetamine-induced cardiomyopathy has become an emerging cause of heart failure in teenage and young adult populations. With its increasingly widespread use for the treatment of attention-deficit/hyperactivity disorder (ADHD) as well as misuse as a “study drug” to increase concentration for educational performance, more patients are at risk of this cardiac manifestation, especially those without knowledge of these risks. It becomes vital to highlight the correlation of amphetamine-dextroamphetamine use and heart failure in order to increase awareness of this condition. This case study highlights the manifestation of tachycardia-induced cardiomyopathy in a 38-year-old female patient with long-standing amphetamine-dextroamphetamine use for refractory narcolepsy. Our patient was evaluated utilizing a thyroid panel, lipid profiles, EKG, Holter monitoring, stress echocardiography, and left heart catheterization, revealing an unremarkable thyroid panel, a left ventricular ejection fraction (LVEF) of 20-25% from 35% to 40% and normal coronary vessels. Amphetamine-dextroamphetamine was discontinued with the introduction of metoprolol, aspirin, and atorvastatin. The mainstay of treatment includes cessation of amphetamine-dextroamphetamine. It is important that physicians continue to recognize the implication of amphetamine-dextroamphetamine in the diagnosis of non-ischemic cardiomyopathy in young patients presenting with heart failure like symptomology and to attain a comprehensive history in order to establish this diagnosis.

## Introduction

Stimulant medications such as amphetamine-dextroamphetamine have known cardiovascular effects by catecholamine-mediated effects such as the induction of tachycardia, vasoconstriction, vasospasm, hypertension, and direct cardiac toxicity [1]. An uncommon but rather important and emerging concern of frequent amphetamine-dextroamphetamine use includes the manifestation of non-ischemic cardiomyopathy possibly secondary to tachycardia leading to heart failure in young adults [2]. This is an occurrence that has become increasingly identified in young adults with heart failure without other compounding risk factors. The importance of this occurrence lies within the medication’s widespread misuse, which places many teens and young adults at great risk for non-ischemic cardiomyopathy and mortality, but also the need for outlining various options for management and follow-up of patients with attention-deficit/hyperactivity disorder (ADHD) who do truly require treatment with this medication. Of note, it becomes imperative that the cardiac implications of the medication are highlighted and presented as the medication’s use is considered. This report presents the case of a 38-year-old female presenting with heart failure due to non-ischemic cardiomyopathy with a long history of amphetamine-dextroamphetamine use.

## Case presentation

The patient is a 38-year-old female with generalized anxiety disorder, narcolepsy, heart failure secondary to non-ischemic dilated cardiomyopathy, mitral and aortic regurgitation, restless leg syndrome, and tobacco use disorder (one pack per day). The workup of her heart failure diagnosis initially manifested from the evidence of possible bi-atrial enlargement, borderline right axis deviation, and sinus tachycardia with occasional premature ventricular complexes (PVCs) noted on the electrocardiogram during her evaluation of post-traumatic stress disorder (PTSD) in 2020 at an out-of-state facility. An ensuing echocardiogram at that time revealed a left ventricular ejection fraction of 35-40% with global hypokinesis, a normal left ventricular and right ventricular chamber diameter, mild right ventricular hypertrophy, and moderate aortic and mitral regurgitation in the absence of pulmonary hypertension. Stress testing throughout that time revealed a metabolic equivalent (METS) of 10.3 (<5 is poor, 6-8 is fair, 9-11 is good, >12 is excellent), a Duke Treadmill score of 0.72 (≥+5 is low risk, −10 to +4 is moderate risk, ≤−11 is high risk), denoting a medium risk for ischemic events. She then underwent myocardial perfusion imaging with gated single-photon emission computed tomography (SPECT) imaging demonstrating global hypokinesis with a left ventricular fraction of 38%. She reportedly had a Holter monitor diary revealing for skipped beats but did not have the Holter monitor results. She had been managed with ivabradine 5 mg for concerns of cardiomyopathy by the preceding cardiology team.

The patient indicated that she often experienced substernal chest pain, longstanding in nature and pressure-based, with intermittent radiation to the right upper extremity. The pain would self-resolve with no identified alleviating factors. The patient reported that her heart rate was usually within the 90s, but without her medications, when emotionally stressed, anxious, or physically active, she would be tachycardic with heart rates as high as the 130s. She did complain of palpitations. She mentioned the use of more pillows in order to sleep due to orthopnea manifesting a few weeks prior to her presentation.

She had a social history including tobacco smoking of one pack per day of cigarettes, and she denied any use of alcohol or recreational drugs such as cocaine or intravenous drug usage. The patient did have children prior and had no miscarriages or recent childbirths. No prior history was noted of post-partum cardiomyopathy, Takotsubo cardiomyopathy, or myocarditis. Her family history did include stroke, hypertension, and diabetes, though she denied any genetic or autoimmune conditions. She had a longstanding history of anxiety and PTSD as well. Regarding her history of narcolepsy, per the patient, she did not respond to standard treatments and was prescribed amphetamine-dextroamphetamine for a number of years for management.

**Figure 1:**
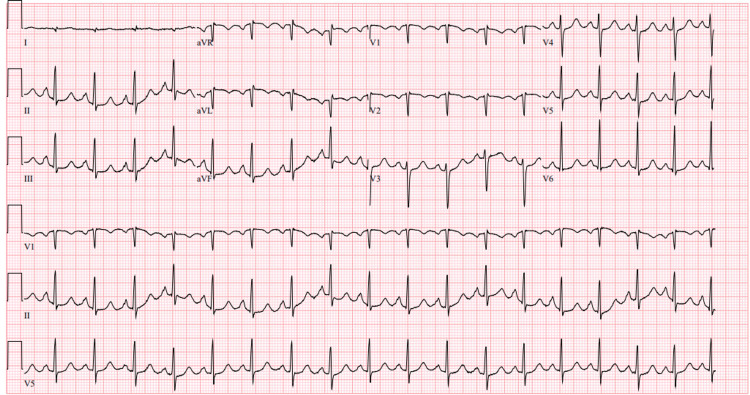
Preliminary ECG evaluation.

Evaluation during the initial consultation for tachycardia, palpitations, and prior documented cardiomyopathy with this treating team revealed tachycardia with a heart rate of 133 and a regular rhythm. No murmurs were appreciated. She had fine rales within the midlung and bases bilaterally and traces of non-pitting bilateral lower extremity edema. ECG revealed sinus tachycardia of 108 beats per minute and suggested bi-atrial enlargement (Figure 1). 

She was prescribed metoprolol succinate 25 mg. The evaluation also included thyroid-stimulating hormone that was unremarkable (1.552 u (IU)/mL, normal 0.350-4.940 u (IU)/mL) and normal lipid panel (low density lipoprotein (LDL) of 50 mg/dL, high density lipoprotein (HDL) of 53 mg/dL, triglycerides of 113 mg/dL, total cholesterol of 121 mg/dL). The brain natriuretic peptide was 16.0 pg/mL (normal 0-100 pg/mL). The ensuing stress transthoracic echocardiogram (TTE) revealed a left ventricular ejection fraction of 20-25%, systolic left ventricular internal dimension (LVID) of 4.4 cm (normal 2.0-4.0 cm), and diastolic LVID of 4.5 cm (normal 3.5-5.6cm), where elevations indicate a higher likelihood of heart failure. The TTE also showed mild to moderate aortic regurgitation, mild mitral regurgitation, and a Duke Treadmill score of 3.42 with an intermediate risk for future cardiac events. She was placed on a 48-hour Holter monitor, which revealed sinus rhythm with rates of 42-106 beats per minute (bpm), averaging 59 bpm, rare ventricular and supraventricular ectopy without significant pauses, and no significant diurnal variations. Subsequently, she underwent a left heart catheterization, which revealed angiographically normal coronary vessels, further reaffirming the diagnosis of non-ischemic cardiomyopathy. Amphetamine-dextroamphetamine had then been discontinued. An automated implantable cardioverter defibrillator (AICD) was not considered, as some patients with methamphetamine-induced cardiomyopathy recover simply with cessation, as further discussed below. Upon one month follow-up with the patient, she did have some improvement in her symptoms.

## Discussion

The medication known as amphetamine-dextroamphetamine has been a pharmaceutical option for treating attention-deficit/hyperactivity disorder for over two decades. It belongs to a class of medications known as stimulants. The medication is composed of a combination of amphetamine salts, including amphetamine and dextroamphetamine, which act by altering catecholamine levels in the nervous system to induce increased neurocognitive stimulation [2], euphoria and increased concentration [1]. The medication can be highly addictive, with tolerance developing quite quickly [1]. The medication has been frequently misused for a number of years by teens and young adults in high school and college without ADHD as a means of increasing neurocognition activation, in efforts to achieve better educational performance [3]. However, as this case highlights, the cardiac changes induced by amphetamine-dextroamphetamine have an implication in the manifestation of non-ischemic cardiomyopathy possibly due to prolonged tachycardia. Thus, with its increasing use, consideration of the dangerous implications of this occurrence in younger patients cannot be overlooked.

Through catecholamine-mediated signaling, the cardiovascular system responds to stimulant use via vasoconstriction, vasospasm tachycardia, and hypertension [4]. Stimulants can induce myocardial fibrosis and changes in myocardial contractility [4,5] with the ensuing onset of dilated or hypertrophic cardiomyopathy.

Patients may present as young teenagers or adults with chest pain, tachycardia, palpitations, or overt signs of heart failure such as orthopnea, paroxysmal nocturnal dyspnea, or peripheral edema without any other known underlying risk factors. It is important to garner a thorough history and evaluation pertaining to the use of any stimulants, any drug use history, recent viral illnesses, family history or personal history of autoimmune conditions or prior underlying or congenital cardiac diseases [6]. It is essential to undergo a comprehensive workup of these patients, including EKG’s, chest x-rays, echocardiograms, stress testing, thyroid function studies, and metabolic profile. The evaluations may reveal a reduction in cardiac function, such as a low left ventricular ejection fraction, global hypokinesis, evidence of cardiac remodeling, and low exercise tolerance for the patient’s age.

The mainstay of treatment is the cessation of amphetamine-dextroamphetamine. Improvements in the patient’s condition occur with cessation, with some studies revealing improvement of the LVEF by 7% in as little as seven days [4]. Other reports have noted benefits in treating the condition as likened to those of other heart failure management methods such as utilizing valsartan or sacubitril, but this has not been widely described in the literature. In some instances, the severity of cardiomyopathy has warranted the need for heart transplantation [1]. The importance of consistent follow-up cannot be understated, with priority placed on addressing adherence to abstinence from the medication. Some studies have recommended periodic transthoracic echocardiogram monitoring for the assessment of improvement or lack thereof subsequent to the cessation of amphetamine-dextroamphetamine [6], though there is no clear delineated timeframe to how often this should be evaluated and may be based on a lack of clinical response to cessation of the medication. There may be a role in consideration of obtaining a baseline echocardiogram when deciding to start amphetamine-dextroamphetamine; however, this is not a current recommended guideline. Additionally, employing a multidisciplinary approach such as involving psychiatric assistance in providing other means of assistance for patients with ADHD requiring treatment, close follow-up for patients placed on amphetamine-dextroamphetamine for concerning cardiac reactivity, as well as therapy for those with a history of misuse of stimulants is a component of management that should be incorporated. A consideration for further study would be the delineation of whether amphetamine-dextroamphetamine should be an absolute contraindication in the context of cardiomyopathy or the reintroduction of the medication as a lower dosage than that originally prescribed, with follow-up of clinical outcomes.

## Conclusions

With the manifestation of cardiomyopathy and heart failure in young patients, amphetamine-dextroamphetamine-induced cardiomyopathy must be considered as a differential diagnosis. It is important to explore any history of the medication’s use during the evaluation of young patient’s presenting with concerns of heart failure given the drug’s widespread prescribed use and misuse. The mainstay of treatment lies within the cessation and avoidance of medication. As part of the therapeutic decision-making process of physicians treating ADHD, the importance of seeking alternative adjunctive strategies to address the prevalence and burden of the use of amphetamine-dextroamphetamine cannot be understated. As this becomes a developing concern, it is important for physicians to continue to recognize the likelihood of this occurrence in their patients in order to be proactive in their management. 
